# Identification of a Fibroblast-Related Prognostic Model in Glioma Based on Bioinformatics Methods

**DOI:** 10.3390/biom12111598

**Published:** 2022-10-30

**Authors:** Haofuzi Zhang, Yutao Huang, Erwan Yang, Xiangyu Gao, Peng Zou, Jidong Sun, Zhicheng Tian, Mingdong Bao, Dan Liao, Junmiao Ge, Qiuzi Yang, Xin Li, Zhuoyuan Zhang, Peng Luo, Xiaofan Jiang

**Affiliations:** 1 Department of Neurosurgery, Xijing Hospital, Fourth Military Medical University, Xi’an 710032, China; 2 Department of Anesthesiology, Xijing Hospital, Fourth Military Medical University, Xi’an 710032, China; 3 Biochemistry and Molecular Biology, College of Life Science, Northwest University, Xi’an 710127, China

**Keywords:** glioma, fibroblast-related genes, risk score, GSEA, prognosis, therapy

## Abstract

Background: Glioma is the most common primary tumor of the central nervous system with a high lethality rate. This study aims to mine fibroblast-related genes with prognostic value and construct a corresponding prognostic model. Methods: A glioma-related TCGA (The Cancer Genome Atlas) cohort and a CGGA (Chinese Glioma Genome Atlas) cohort were incorporated into this study. Variance expression profiling was executed via the “limma” R package. The “clusterProfiler” R package was applied to perform a GO (Gene Ontology) analysis. The Kaplan–Meier (K–M) curve, LASSO regression analysis, and Cox analyses were implemented to determine the prognostic genes. A fibroblast-related risk model was created and affirmed by independent cohorts. We derived enriched pathways between the fibroblast-related high- and low-risk subgroups using gene set variation analysis (GSEA). The immune infiltration cell and the stromal cell were calculated using the microenvironment cell populations-counter (MCP-counter) method, and the immunotherapy response was assessed with the SubMap algorithm. The chemotherapy sensitivity was estimated using the “pRRophetic” R package. Results: A total of 93 differentially expressed fibroblast-related genes (DEFRGs) were uncovered in glioma. Seven prognostic genes were filtered out to create a fibroblast-related gene signature in the TCGA-glioma cohort training set. We then affirmed the fibroblast-related risk model via TCGA-glioma cohort and CGGA-glioma cohort testing sets. The Cox regression analysis proved that the fibroblast-related risk score was an independent prognostic predictor in prediction of the overall survival of glioma patients. The fibroblast-related gene signature revealed by the GSEA was applicable to the immune-relevant pathways. The MCP-counter algorithm results pointed to significant distinctions in the tumor microenvironment between fibroblast-related high- and low-risk subgroups. The SubMap analysis proved that the fibroblast-related risk score could predict the clinical sensitivity of immunotherapy. The chemotherapy sensitivity analysis indicated that low-risk patients were more sensitive to multiple chemotherapeutic drugs. Conclusion: Our study identified prognostic fibroblast-related genes and generated a novel risk signature that could evaluate the prognosis of glioma and offer a theoretical basis for clinical glioma therapy.

## 1. Introduction

Tumors of the central nervous system (CNS) are a devastating group of diseases. Glioma is a primary malignant tumor derived from glial cells, accounting for approximately 75% of the malignant tumors of the CNS [1,2]. It originates from supporting glial cells or their precursors in the brain. The World Health Organization (WHO) classified gliomas as grade I–IV according to cell morphology, malignancy, and pathogenicity. Low-grade gliomas are characterized by cell morphology, which can have local effects and will not spread in the brain. High-grade gliomas are malignant and can spread to the whole brain tissue. Clinically, gliomas are usually divided into low-grade glioma (LGG) and glioblastoma (GBM). LGG grows slowly and can be removed by surgery, and the prognosis of patients is relatively good [3]. However, GBM is a grade IV malignant glioma, accounting for 50% of adult primary brain tumors [4,5], and one of the most difficult human cancers to treat. The extensive invasion of tumor cells into the surrounding functional brain tissue prevents the complete surgical removal of the tumor [6]. Even with surgical resection and chemotherapy, the overall prognosis of GBM patients is still poor, and the median survival time is approximately 14–15 months [7].

Although the overall prognosis of patients with GBM is poor, there are still significant differences in the prognosis of these patients [8]. Therefore, it is necessary to find biomarkers to improve the prognosis of patients with GBM. Intertumoral heterogeneity refers to the difference between tumor cells and a series of supporting cells, immune cells, and stromal cells that provide a comfortable environment for the development and growth of tumor cells [9]. Intercellular heterogeneity includes a range of non-tumor cells (invasive and resident immune-related cells, stromal cells, and other non-tumor glial cells), the extracellular matrix (ECM), and other constituents relevant to the tumor microenvironment (TME) [10].

The dynamic interaction between tumor cells and the TME plays an important role in the occurrence, progression, and metastasis of cancer, as well as in anticancer efficacy and drug resistance [11,12]. Tumor-associated fibroblasts (TAFs), as the most important host cells in TME, can promote tumorigenesis and enhance the invasiveness of cancer cells. They can also induce chronic inflammation by producing the proinflammatory cytokines responsible for immune tolerance [13]. Under normal conditions, fibroblasts maintain the structural integrity of tissues by synthesizing the extracellular matrix (ECM) of connective tissue [14]. In the TME, TAFs have been shown to secrete growth factors, inflammatory ligands, and extracellular matrix proteins, which can promote cancer cell proliferation, therapeutic resistance, and immune rejection [15].

With the rapid development of The Cancer Genome Atlas (TCGA) and the Chinese Glioma Genome Atlas (CGGA), this study aims to find fibroblast-related genes with prognostic value and design prognostic models to predict the sensitivity of immunotherapy and drug therapy in glioma patients.

## 2. Materials and Methods

### 2.1. Data Source

We collected RNA sequencing (HTSeq-FPKM) data and corresponding clinical information of 5 normal samples and 644 glioma samples from the TCGA database (https://www.cancer.gov/tcga, accessed on 7 February 2022), of which 635 glioma samples with duration of survival information were used for prognostic analysis (Table S1). The RNA sequencing (HTSeq-FPKM) data of 657 glioma samples with survival information were also mined from the CGGA database [16] as a validation cohort for the prognostic model (Table S2). A total of 2784 fibroblast-related genes (FRGs) were extracted from the molecular signatures database (MsigDB) (version 7.5.1) (http://www.gsea-msigdb.org/gsea/msigdb/, accessed on 6 February 2022) by using the keyword “fibroblast”. The data processing pipeline is shown in Figure S1.

### 2.2. Certification of Differentially Expressed Fibroblast-Related Genes (DEFRGs)

The variance expression profiling between the normal and glioma specimens in the TCGA cohort was implemented via the “limma” R package (version 3.52.2) [17]. The cut-off criterion was |log2FoldChange (FC)| > 2 and adjusted *p*-value < 0.05. Using the Benjamini–Hochberg method [18], *p*-values were adjusted to control for the false discovery rate (FDR). The differentially expressed fibroblast-related genes (DEFRGs) were authenticated by overlapping the differentially expressed genes (DEGs) with 2784 FRGs from the MsigDB using a Venn diagram [19].

### 2.3. Functional Enrichment Analysis

A GO (Gene Ontology) enrichment analysis was implemented using the “clusterProfiler” R package (version 4.4.4) [20]. The standard for significance was an adjusted *p*-value of <0.05, the FDR correction was adopted to adjust the *p*-value for multiple tests. The GO analysis was composed of biological processes (BP), cellular components (CC), and molecular functions (MF).

### 2.4. Establishment and Validation of the Fibroblast-Related Risk Score Model

To preliminary screen prognostic genes, we first incorporated DEFRGs into the survival analysis in the TCGA cohort and plotted the corresponding K–M curves. Genes with *p*-value < 0.001 for survival difference between the high- and low-expression group were enrolled in the univariate Cox analysis. The 635 glioma patients with survival information in the TCGA cohort were segmented randomly into a training cohort (445 cases) and a testing cohort (190 cases) at a ratio of 7:3. The univariate Cox analysis was applied to appraise the genes that were remarkably linked to the survival of the glioma patients in the training set (*p*-value < 0.001). The acquired genes were then included in a least absolute shrinkage and selection operator (LASSO) logistic regression to filter out the key genes. Subsequently, the acquired key genes were submitted to multivariate regression analysis with a stepwise function to uncover the fibroblast-related prognostic genes and the corresponding coefficients of the prognostic genes. The risk score calculating formula was: Riskscore = β1X1 + β2X2 +…+ βnXn. In this formula, β refers to the regression coefficient and X represents the expression value of the prognostic genes.

To stratify patients into high- and low-risk groups, the median of the risk score was determined. The K–M curve was drawn to estimate the overall survival (OS) distinction between two risk fibroblast-related subgroups using the “survminer” R package (version 0.3.1) [21]. Receiver operating characteristic (ROC) curves were generated with the “survival ROC” package (version 1.0.3) [22] in R to evaluate the validity of the prognostic model. Furthermore, the risk plot was developed via the “pheatmap” package (version 1.0.10) [23] in R. The expression of prognostic genes in the high- and low-risk groups was drawn in the corresponding heatmap. We deployed the CGGA cohort as the external validation cohort. The above procedure was carried out in the training cohort, testing cohort, and validation cohort, respectively, to create and affirm the fibroblast-related risk score model.

### 2.5. Correlation of Risk Score and Clinical Characteristics

We first plotted a heatmap to show the distribution of risk scores for clinical characteristics. The correlation between risk score and clinical characteristics was then assessed using chi-squared tests [24].

### 2.6. Independent Prognostic Analysis and Construction of a Nomogram

Cox regression (univariate and multivariate) analyses were executed to recognize independent predictors of glioma OS in the training cohort via the “survminer” R package (version 0.3.1) [21]. Subsequently, a nomogram composed of the independent predictors of glioma OS was developed using the “rms” R package (version 5.1-2) [25]. Calibration curves (1-, 3-, and 5-year) were drawn to confirm the accuracy of the nomogram.

### 2.7. Gene Set Enrichment Analysis

Gene Set Enrichment Analysis (GSEA) between the low- and high-risk groups, and between glioma patients with survival time greater than 5 years and glioma patients with survival time less than 5 years was implemented using the “clusterProfiler” R package (version 4.4.4) based on differential analysis conducted via the “limma” R package (version 3.52.2) [17]. The threshold for significantly enriched items and pathways was |NES| > 1, NOM *p*-value < 0.05, and q-value < 0.25. The scores for the significantly enriched items and pathways in each sample were further calculated using the ssGSEA approach, and the corresponding clustering was performed.

### 2.8. Assessment of Immune Infiltration Cell and Stromal Cell, and Immunotherapy Response between Two Fibroblast-Related Risk Subgroups

The enriched abundance of the immune cells, fibroblasts, and endothelial cells in each sample was computed with the microenvironment cell populations-counter (MCP-counter) algorithm [26]. A box plot was drawn to visualize the differences between the low- and high-risk groups. The correlations between 10 types of cells and the risk score were also calculated. The expression of the routine immune checkpoint molecules in the high- and low-risk groups were further analyzed and compared. The SubMap algorithm was utilized to assess the sensitivity of immunotherapy between the low- and high-risk groups [27].

### 2.9. Subsection Evaluation of Chemotherapy Sensitivity between Two Fibroblast-Related Risk Subgroups

The sensitivity of chemotherapeutic drugs was obtained from the GDSC database [28]. The half-maximum inhibitory concentration (IC50) was estimated using the “pRRophetic” R package (version 0.5) [29] to predict the response to chemotherapeutic drugs in the high- and low-risk groups.

### 2.10. Methylation Analysis of Prognostic Genes

We first downloaded methylation data of glioma samples from the TCGA database. We then analyzed the Spearman correlation between the expression of seven prognostic genes and the beta value of the corresponding cg site. Corresponding scatter plots were plotted. The criterion for a significant correlation was *p*-value < 0.05.

### 2.11. Correlation Analysis between Prognostic Gene Expression and CNV

We downloaded copy number variation (CNV) data of glioma samples from the cBioPortal database (https://www.cbioportal.org/, accessed on 15 February 2022). To investigate whether the expression of the prognostic gene was related to CNV, we performed a Spearman correlation analysis between the CNV and the expression of the prognostic gene. Corresponding scatter plots were mapped. The criterion for a significant correlation was a *p*-value of <0.05.

### 2.12. Antibodies

Antibodies against CDK4 (#GB11238-2, rabbit pAb) and SLC2A3 (#GB11294, rabbit pAb) were obtained from Servicebio (Wuhan, China). Antibodies against NEGR1 (#bs-11095R, rabbit pAb) and MEX3A (#bs-18820R, rabbit pAb) were obtained from Bioss ANTIBODIES (Beijing, China). ANGPTL2 (#12316-1-AP, rabbit pAb), PBK (#16110-1-AP, rabbit pAb), and TMEM100 (#25581-1-AP, rabbit pAb) were obtained from Proteintech (Rosemont, IL, USA). For immunohistochemistry, HRP-conjugated goat anti-rabbit/mouse IgG H&L (#PV-6000D) secondary antibodies (ZSGB-bio, Beijing, China) were used.

### 2.13. Immunohistochemistry (IHC)

The slides were retrieved using the heat-induced epitope retrieval method and then blocked in 5% goat serum (#SP-9001, ZSGB-bio, Beijing, China) for 10 min. Next, the primary antibody was added and incubated with gentle agitation overnight at 4 °C. After washing with PBS three times, the secondary antibody working solution was subsequently added and incubated for 10 min at room temperature. The slides were washed with PBS three times, and then the streptavidin/HRP working solution (#SP-9001, ZSGB-bio, Beijing, China) was added and incubated for 10 min at room temperature. After washing with PBS three times, fresh DAB working solution (#SP-9001, ZSGB-bio, Beijing, China) was added and incubated for 5 min at room temperature. The slides were washed, hematoxylin-stained, and finally observed under inverted bright field microscopy.

### 2.14. Statistical Analysis

All bioinformatics analyses were conducted using the R programming language, and the data from different groups were compared using the Wilcoxon test. If not specified above, a *p*-value less than 0.05 was considered statistically significant.

## 3. Results

### 3.1. DEFRGs in Glioma

**Figure 1:**
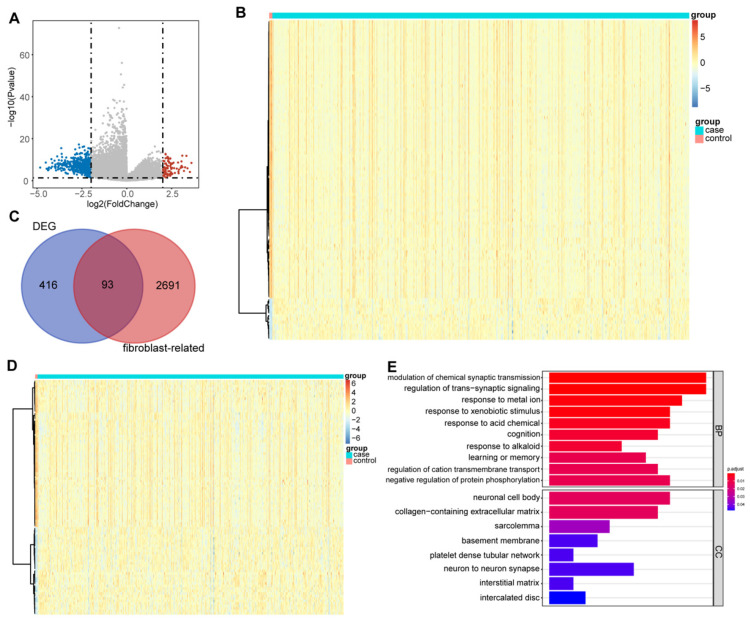
Identification of 93 DEFRGs: (**A**) volcano plot of DEGs between the glioma and normal samples from the TCGA cohort, blue dots: down-regulation and red dots: up-regulation; (**B**) heatmap of top 100 DEGs between the glioma and normal samples, each small square represents one sample; (**C**) Wayne diagram of DEFRGs; (**D**) heatmap of the expression of DEFRGs; and (**E**) top 10 GO items in each category.

In the TCGA cohort, a total of 509 DEGs between the glioma and normal tissues, including 88 up-regulated genes and 421 down-regulated genes in glioma, were excavated according to the threshold value (Table S3 and Figure 1A). The top 100 DEGs are displayed in Figure 1B. We then obtained 2784 FRGs from the MsigDB (Table S4). Hence, 93 DEFRGs between the glioma and normal samples were certificated by crossing the DEGs with the FRGs (Figure 1C, Table S4). The expression of DEFRGs was drawn into a heatmap (Figure 1D). To probe the function and involved pathways of the DEFRGs, we carried out a GO analysis. In total, 74 GO items, including 66 BP items and 8 CC terms, were derived (Table S5). The top 10 items in each classification are presented in Figure 1E. We noted that the DEFRGs were associated with the modulation of chemical synaptic transmission, the positive regulation of fibroblast proliferation, and neuron-related biological processes.

### 3.2. The Fibroblast-Related Risk Score Model Based on DEFRGs

**Figure 2:**
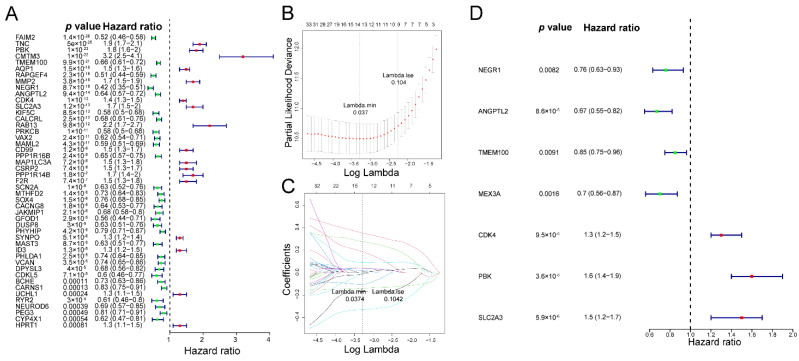
Identification of fibroblast-related prognostic genes: (**A**) forest map of univariate Cox regression analysis; (**B**,**C**) lasso logistic regression coefficient penalty diagram; and (**D**) forest map of multivariate Cox regression analysis.

**Figure 3:**
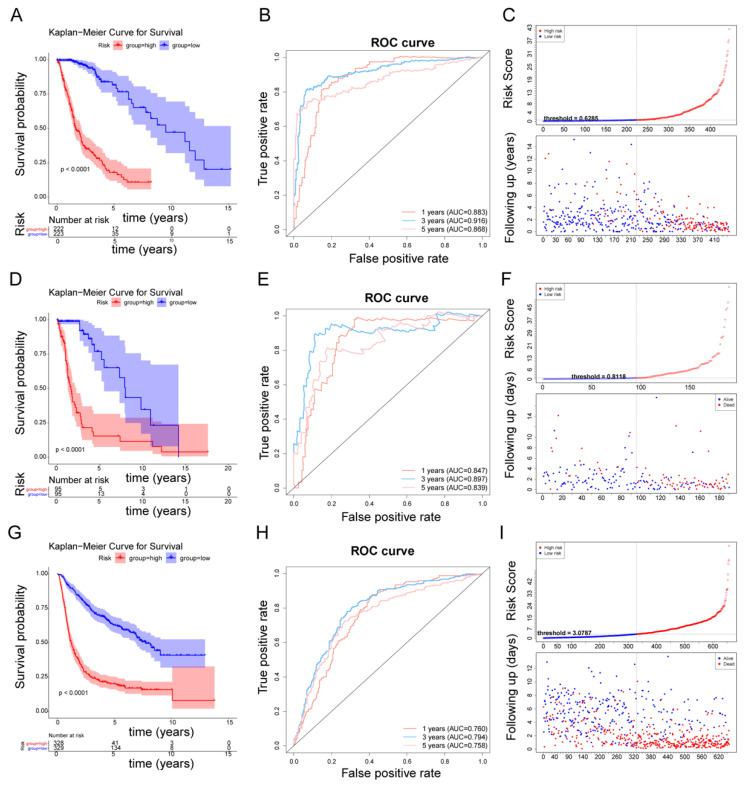
Construction and validation of the fibroblast-related risk score model: (**A**) Kaplan–Meier survival curve of high- and low-risk groups in the training cohort; (**B**) ROC curve of 1, 3, and 5 years in the training cohort; (**C**) risk curve of the high- and low-risk groups in the training cohort; (**D**) Kaplan–Meier survival curve of the high- and low-risk groups in the testing cohort; (**E**) ROC curve of 1, 3, and 5 years in the testing cohort; (**F**) risk curve of the high- and low-risk groups in the testing cohort; (**G**) Kaplan–Meier survival curve of the high- and low-risk groups in the validation cohort; (**H**) ROC curve of 1, 3, and 5 years in the validation cohort; and (**I**) risk curve of the high- and low-risk groups in the validation cohort.

We conducted a survival analysis based on the expression of 93 DEFRGs and the survival data of glioma patients in the TCGA cohort. As revealed in Table S6 and Figure S2, a total of 57 DEFRGs that were extremely associated with glioma survival (*p*-value < 0.001) were selected. Next, 635 glioma patients in the TCGA cohort were separated into two cohorts: a training cohort with 445 patients and a testing cohort with 190 patients. A univariate Cox analysis was then performed with the uncovering of prognosis-related genes on the basis of 57 DEFRGs in the training cohort. Of the 57 DEFRGs, 47 were identified as being remarkably associated with the patients’ survival in the training cohort (*p*-value < 0.001) (Figure 2A, Table S7). Subsequently, the 47 genes were further submitted to a LASSO regression analysis. A total of 13 key genes (NEGR1, ANGPTL2, TMEM100, SOX4, BCHE, MEX3A, TNC, CD99, AQP1, CMTM3, CDK4, SLC2A3, and PBK) were selected using the LASSO regression algorithm (Figure 2B–C). Next, the 13 aforementioned genes were enrolled in a stepwise multivariate Cox regression analysis. Seven DEFRGs (NEGR1, ANGPTL2, TMEM100, MEX3A, CDK4, SLC2A3, and PBK) were recognized as prognostic genes (Figure 2D, Table S8), and each regression coefficient was derived. We hence developed a fibroblast-related risk score model with this formula: risk score = −0.26977399 × expression of NEGR1 + –0.40452456 × expression of ANGPTL2 + −0.16436047 × expression of TMEM100 + −0.35367967 × expression of MEX3A + 0.28419244 × expression of CDK4 + 0.48233077 × expression of PBK + 0.37747596 × expression of SLC2A3. The patients were then divided into two risk subgroups (high- and low-risk), depending on the median value of the risk score in the training cohort and the testing cohort, respectively. The K–M survival curves showed that patients with a higher risk had notably worse survival rates than those with a lower risk (Figure 3A,D). The area under the curve (AUC) values of 1-, 3-, and 5-year overall survival (OS) in the training cohort were 0.883, 0.916, and 0.868, respectively, revealing excellent accuracy (Figure 3B). Meanwhile, the AUC values of 1-, 3-, and 5-year OS in the testing cohort were 0.847, 0.897, and 0.839, respectively, further suggesting the good predictive power of the risk signature (Figure 3E). The risk curve revealed that, as the risk score increased, patients confronted a higher risk of death (Figure 3C,F). The expression of seven prognostic genes in two risk subgroups are presented in Figure S3A,B. The heatmap results suggested that CDK4, PBK, and SLC2A3 were highly expressed in the patients with a higher risk. NEGR1, MEX3A, ANGPTL2, and TMEM100 were highly expressed in the patients with a lower risk.

### 3.3. Affirmation of the Fibroblast-Related Risk Model Via External Cohort

An independent cohort with 657 glioma patients from the CGGA cohort was deployed to externally verify the fibroblast-related model accuracy. In accordance with the TCGA cohort, patients with a higher risk endured worse OS (Figure 3E) in the validation set. The AUC values in predicting prognosis were 0.760 (1-year), 0.794 (3-year), and 0.758 (5-year) (Figure 3H). Figure 3I presents the survival status based on the patients with increased risk scores, and the heatmap showed a comparable result to the training set (Figure S3C). These results further affirm the applicability of the FRG-related risk score model in assessing the 1-, 3-, and 5-year survival of glioma patients.

### 3.4. Correlation between the FRG-Related Gene Signature and Clinicopathological Characteristics

To explore the role of the fibroblast-related risk signature in glioma progression, we researched the relevance between risk score and clinical characteristics. The distribution of the risk score for clinical characteristics in the training cohort, testing cohort, and validation cohort is revealed in Figure S3A–C. As shown in Table 1, the risk score was correlated with age, isocitrate dehydrogenase (IDH), grade, and methylation of O6-methylguanine-DNA methyltransferase (MGMT) status in the training cohort and testing cohort. Meanwhile, we noted that the risk score was correlated with age, IDH, grade, and histology in the validation cohort.

### 3.5. Fibroblast-Related Risk Signature Is an Independent Predictor for Glioma Prognosis

**Figure 4:**
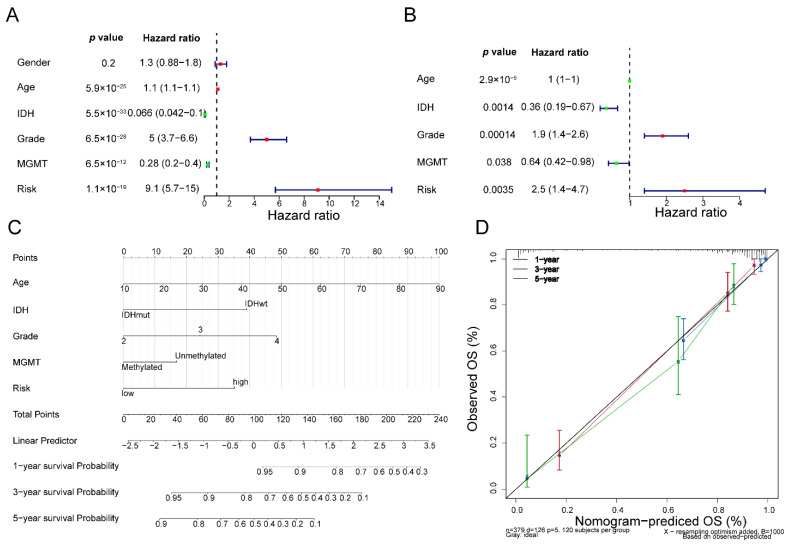
The fibroblast-related nomogram model: (**A**) forest map of univariate Cox regression analysis; (**B**) forest map of multivariate Cox regression analysis; (**C**) nomogram for predicting survival with independent prognostic factors; and (**D**) calibration curve for nomogram.

To further probe the independent prognostic value of the fibroblast-related risk signature, we enrolled clinicopathological factors (age, gender, IDH, grade, and MGMT) along with the fibroblast-related risk score into the Cox analysis. The univariate analysis indicated that the *p*-values of age, IDH, grade, MGMT, and risk score were smaller than 0.05 (Figure 4A), and multivariate Cox regression analyses were conducted. The multivariate analysis forest plot proved that the fibroblast-related risk score was an independent predictor for glioma prognosis (Figure 4B). Additionally, age, IDH, grade, and MGMT were also authenticated as independent prognostic factors (Figure 4B). Therefore, we developed a nomogram employing independent prognostic factors, including age, IDH, grade, MGMT, and risk score, to assess the 1-, 3-, and 5-year OS of patients (Figure 4C). The C index of the fibroblast-related nomogram was 0.878. The calibration plots proved that the nomogram performed quite well in forecasting the 1-, 3-, and 5-year survival probability for glioma patients (Figure 4D).

### 3.6. GSEA between the Two Fibroblast-Related Risk Subgroups

**Figure 5:**
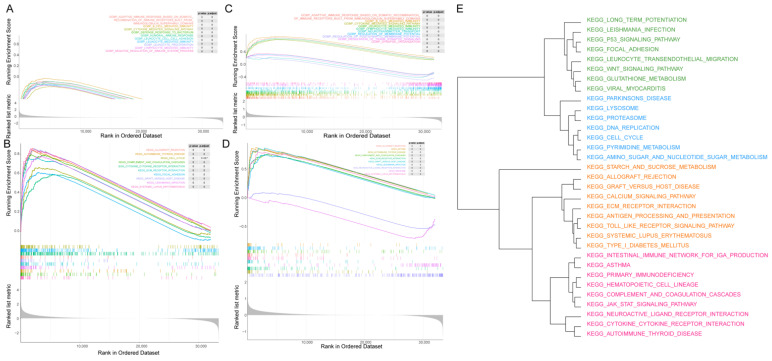
GSEA results related to the prognosis of patient: (**A**) top 10 GO items enriched by high- and low-risk groups; (**B**) top 10 KEGG items enriched by high- and low-risk groups; (**C**) top 10 GO items enriched by patients with survival times longer and shorter than 5 years; (**D**) top 10 KEGG items enriched by patients with survival times longer and shorter than 5 years; and (**E**) clustering tree of shared KEGG pathways.

To probe the possible mechanism for the distinction in the prognosis between the two fibroblast-related risk subgroups, GSEA was applied to profile the dynamics of the biological pathways and processes between the two fibroblast-related risk subgroups. A total of 981 GO items and 38 KEGG (Kyoto Encyclopedia of Genes and Genomes, https://www.kegg.jp/, accessed on 10 February 2022) pathways were enriched (Table S9). The top 10 GO items and KEGG pathways are exhibited in Figure 5A,B. Multiple immune-related biological processes and pathways, and cell cycle-related pathways were related to the risk score. Further, GSEA was performed to determine the different biological functional states of glioma patients with survival times longer and shorter than 5 years. A total of 983 GO items and 37 KEGG pathways were enriched between the glioma patients with survival times longer and shorter than 5 years (Table S10). The top 10 GO items and KEGG pathways are exhibited in Figure 5C,D. Similarly, a number of immune-related processes and pathways, and cell cycle-related pathways were enriched between patients who survived more than 5 years and those who survived less than 5 years. A total of 758 common GO items and 33 common KEGG pathways were identified by overlapping the GSEA results from the above two comparisons (Table S11). The scores for the common enriched items and pathways in each sample were further calculated using the ssGSEA method, and the corresponding clustering was performed. Figure S4 shows the clustering of the GO items. Meanwhile, 33 KEGG pathways were clustered into four clusters, including metabolism-related, immune-related, and cancer-related pathways (Figure 5E). These data suggest that the above pathways and processes played an important role in influencing the prognosis of the high- and low-risk groups.

### 3.7. Immune Infiltration Cell, Stromal Cell, and Immunotherapy Response between the Two Fibroblast-Related Risk Subgroups

**Figure 6:**
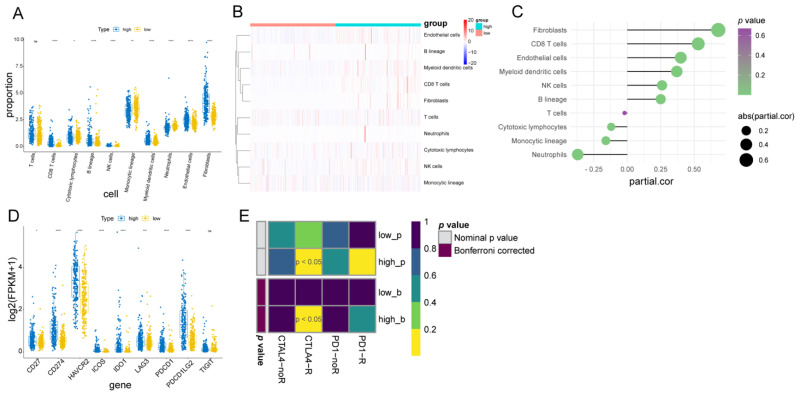
Tumor microenvironment and immunotherapy analysis of high- and low-risk groups: (**A**) box plot to show the difference in immune cells and stromal cells between the high- and low-risk groups; (**B**) heatmap of cell contents calculated using MCP-counter; (**C**) lollipop chart to show the correlation between risk score and immune cells and stromal cells; (**D**) box plot of immune checkpoints in high- and low-risk groups; and (**E**) SubMap algorithm result to predict the response probability of high- and low-risk groups to immunotherapy.

Since immune-related pathways were linked to the two fibroblast-related risk subgroups, we next performed a variance analysis of infiltrating cells between the two risk subgroups. The MCP-counter results show that the relative abundance of CD8 T cells, B lineage, NK cells, myeloid dendritic cells, endothelial cells, and fibroblasts was significantly higher in the high-risk group compared with the low-risk group (Figure 6A,B). Meanwhile, the relative abundance of monocytic lineage, cytotoxic lymphocytes, and neutrophils was significantly lower in the high-risk group compared with the low-risk group (Figure 6A,B). The correlation analysis revealed that the risk score was significantly associated with differential infiltrating cells between the low- and high-risk groups (Figure 6C). Hence, we speculated that there were notable distinctions in the tumor microenvironments between the two fibroblast-related risk subgroups.

We then compared the expression of immune checkpoints between the high- and low-risk groups. The results show that IDO1, PD-L1 (CD274), PD-L2 (PDCD1LG2), TIM-3 (HAVCR2), PD-1 (PDCD1), LAG3, ICOS, and CD27 were highly expressed in the high-risk group compared with the low-risk group (Figure 6D), implying that the high-risk group was more sensitive to immunotherapy. Therefore, we used SubMap to predict the response probability to immunotherapy in the high- and low-risk groups. The result shows that the high-risk group was more likely to be more sensitive to anti-CTLA4 therapy (Figure 6E, Bonferroni-corrected *p*-value < 0.05).

### 3.8. DEFRGs in Glioma

**Figure 7:**
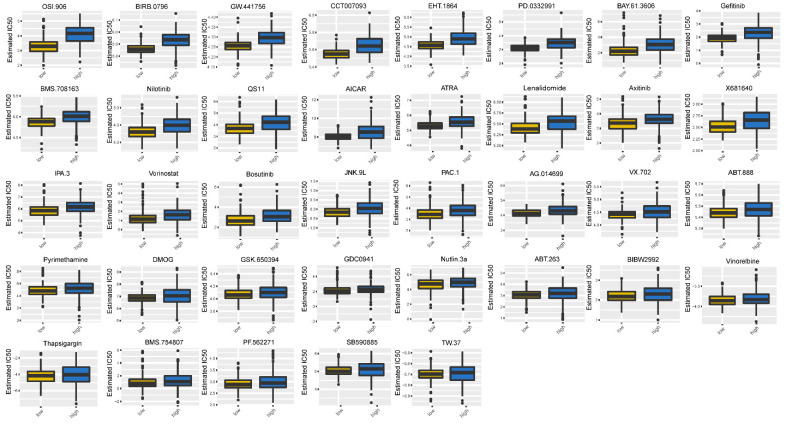
Chemotherapy drug sensitivity in high- and low-risk groups. * *p* < 0.05, ** *p* < 0.01, *** *p* < 0.001, **** *p* < 0.0001.

Next, we proceeded to analyze the chemotherapeutic drug sensitivity of the high-risk and low-risk groups. As revealed in Figure 7, the IC50 values for 37 drugs for the patients with a lower risk were significantly lower than for the patients with a higher risk, indicating that the fibroblast-related low-risk patients were more sensitive to chemotherapeutic drugs, including gefitinib, nilotinib, lenalidomide, axitinib, vorinostat, bosutinib, pyrimethamine, vinorelbine, and thapsigargin.

### 3.9. Methylation and CNV Analysis of Prognostic Genes

**Figure 8:**
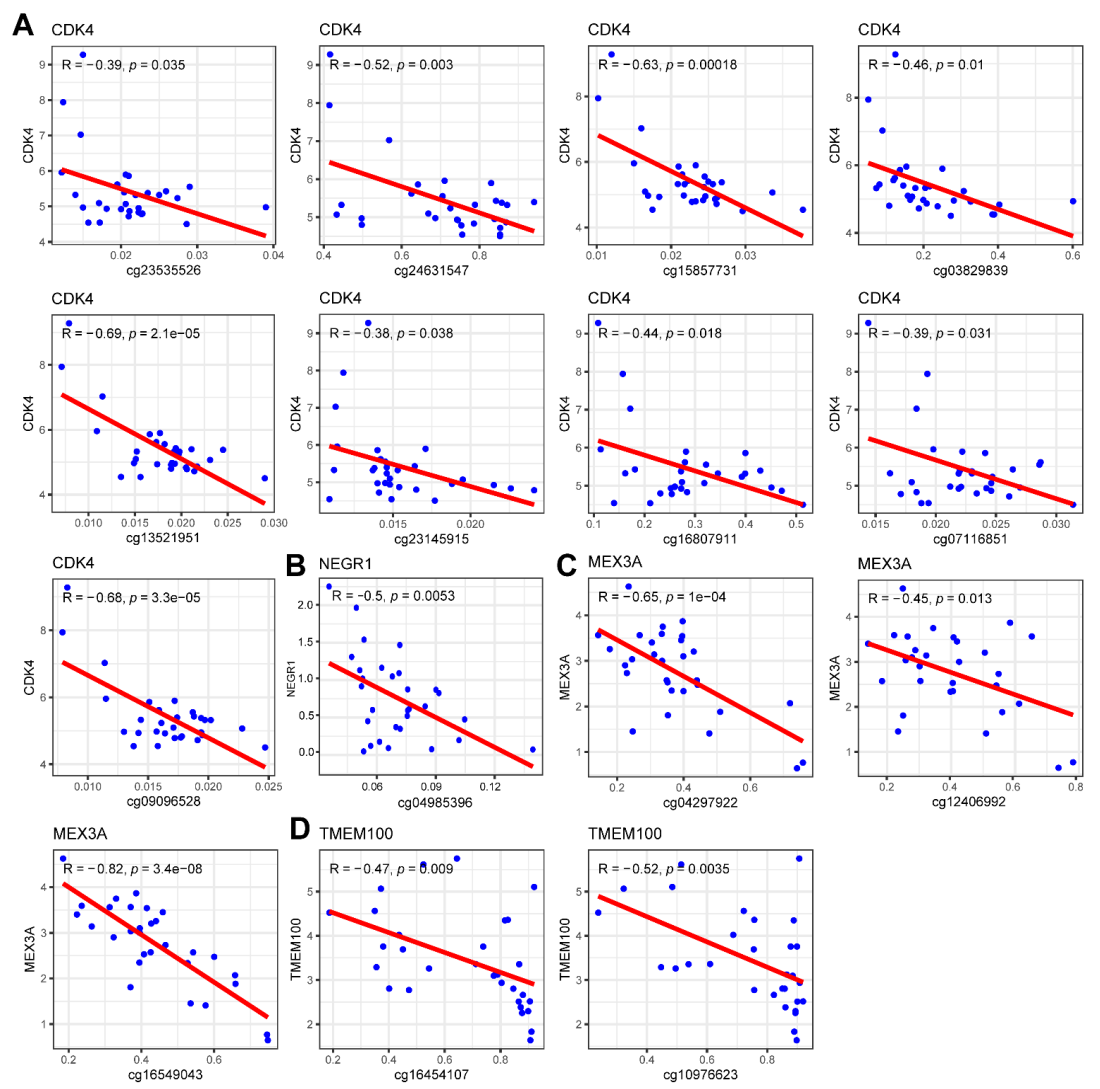
Correlation scatter plot of the significantly negative correlations between the expression of prognostic genes and different cg sites: (**A**) correlation scatter plot of CDK4 expression and its corresponding cg sites; (**B**) correlation scatter plot of NEGR1 expression and its corresponding cg sites; (**C**) correlation scatter plot of MEX3A expression and its corresponding cg sites; and (**D**) correlation scatter plot of TMEM100 expression and its corresponding cg sites.

**Figure 9:**
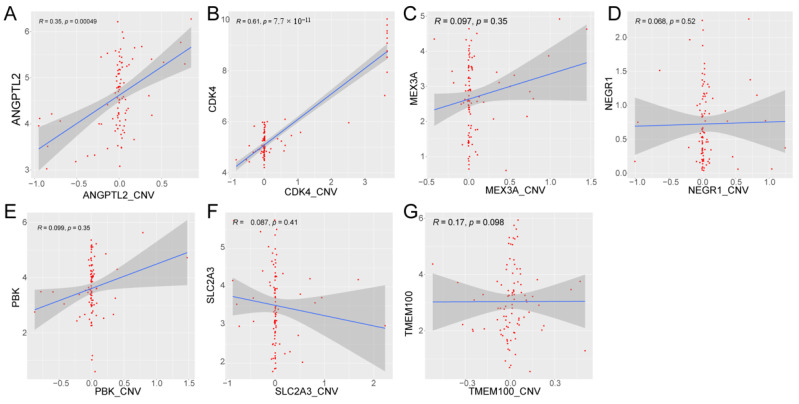
Correlation between prognostic gene expression and CNV: (**A**) correlation scatter plot of ANGPTL2 expression and CNV; (**B**) correlation scatter plot of CDK4 expression and CNV; (**C**) correlation scatter plot of MEX3A expression and CNV; (**D**) correlation scatter plot of NEGR1 expression and CNV; (**E**) correlation scatter plot of PBK expression and CNV; (**F**) correlation scatter plot of SLC2A3 expression and CNV; and (**G**) correlation scatter plot of TMEM100 expression and CNV.

To further explore the mechanism of the aberrant expression of prognostic genes in glioma, we first analyzed the correlation between the expression of prognostic genes and the corresponding methylation sites (Table S12, Figure 8, and Figure S5). DNA methylation is an epigenetic modification of genes that mainly leads to gene transcription silencing. We did not detect ANGPTL2-associated methylation site data. The significantly negatively correlation between prognostic genes and methylation sites is shown in Figure 8. The expression of CDK4 was negatively correlated with nine methylation sites, including cg23535526, cg24631547, cg15857731, cg03829839, cg13521951, cg23145915, cg16807911, cg07116851, and cg09096528. The expression of NEGR1 was negatively correlated with the methylation of cg04985396. The expression of MEX3A was negatively correlated with three methylation sites, including cg04297922, cg12406992, and cg16549043. The expression of TMEM100 was negatively correlated with two methylation sites, including cg16454107 and cg10976623. PBK and SLC2A3 were not significantly negatively correlated with the corresponding methylation sites (Figure S5E,F). The above results imply that the expression of CDK4, NEGR1, MEX3A, and TMEM100 may be regulated by DNA methylation in glioma. Finally, we analyzed the correlation between the expression of prognostic genes and CNV. The results show that the expression of CDK4 and ANGPTL2 was significantly associated with CNV (Figure 9), indicating that the expression of CDK4 and ANGPTL2 in glioma may be affected by CNV.

### 3.10. Validation of Prognostic Gene Expression

**Figure 10:**
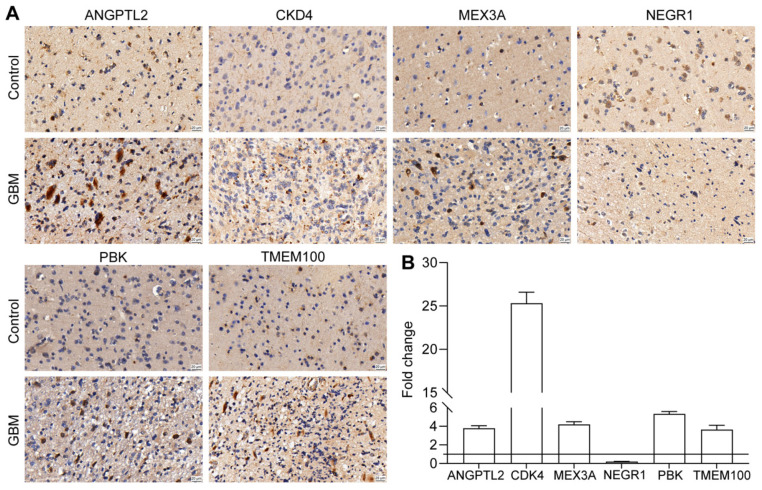
Validation of the expression of prognostic genes: (**A**) IHC images of the protein expression of significant prognostic genes. Bronze area shows the expression of the detected protein (40× magnification, bar = 20 μm); and (**B**) quantification of the activity of horseradish peroxidase. Fold change is the ratio of signal density between the control and GBM samples.

From the transcriptome data analysis results in the public database (Table S3), it was found that CDK4, PBK, ANGPTL2, TMEM100, and MEX3A were significantly up-regulated in the glioma samples compared with the normal samples, whereas NEGR1 and SLC2A3 were down-regulated in the glioma samples at the mRNA level. Furthermore, we used clinical GBM samples and control samples for the immunohistochemical detection of the protein expression of seven prognostic genes at the protein level, except SLC2A3 (not quite significant at the protein level), all reaching the same conclusion as the public database (Figure 10A,B).

## 4. Discussion

The occurrence, development, and prognosis of cancer are closely related to the immune function of the body. The tumor immune escape mechanism is one of the most influential risk factors in the occurrence of many types of cancer. The role of the TME in the process of tumor immune escape has also attracted increasing attention [30], with non-tumor components in the immune system, especially TAFs in glioma, becoming the focus [31]. TAFs are a group of cells of different cell origins that can be derived from a variety of cells, such as static fibroblasts, epithelial cells, and endothelial cells [32]. As a key factor, TAFs produce a variety of ECM proteins and regulatory molecules, and construct a microenvironment conducive to tumor initiation, angiogenesis, and tumor invasiveness [33]. A deeper understanding of the biological function of TAFs will help us to better appreciate how TAFs affect the dynamic complexity and functional plasticity of the TME in glioma. At present, with the TCGA and CGGA databases, we can perform comprehensive bioinformatics analyses to find the fibroblast-related genes connected to glioma prognosis.

To screen out the genes related to glioma, we downloaded the TCGA database glioma transcriptome data and performed a differential analysis on normal and disease samples. A total of 509 distinctly expressed genes were obtained that may contain genes with a significant impact on the occurrence and development of glioma. At the same time, we found 2784 FRGs in the MSigDB. Taking the intersection of the above two gene sets, a total of 93 DEFRGs distinctly expressed in glioma were obtained. We found that the DEFRGs were associated with chemical synaptic transmission and fibroblast proliferation. It has been proven in previous studies that neuron and glioma interactions include electrochemical communication through neuron–glioma synapses [34], and that the fibroblast growth factor receptor (FGFR) gene can be used as a target to intervene in some glioblastoma subsets [35]. However, it is necessary to further explore the potential complex mechanism of DEFRGs in gliomas and gliomagenesis.

Next, we conducted an overall survival (OS) analysis based on the expression level of 93 DEFRGs and the survival information of glioma patients, obtaining 57 genes that are extremely associated with the survival of glioma patients. To improve the accuracy and relevance of this study, we continued to use the univariate Cox regression analysis, LASSO regression analysis, and multivariate Cox regression analysis to identify prognostic genes. We finally screened seven genes as the prognostic factors of this study. It has been reported that CDK4 knockdown impedes the colony formation and cell proliferation of glioma [36], and that PBK and SLC2A3 could be a potential prognostic factor and therapeutic target for GBM treatment [37,38]. In addition, the knockdown of ANGPTL2 and MEX3A reduces the proliferative and invasive abilities of glioma cells [39,40]. The role of NEGR1 and TMEM100 in glioma has not yet been reported. We found for the first time that they were related to the prognosis of glioma.

We evaluated the risk score model in the TCGA cohort and determined the effectiveness of the risk score model, since the area under the curve (AUC) was greater than 0.80. To analyze the correlation between risk score and clinical characteristics, we presented the available clinical data in the dataset, combined the risk score with the clinical data of patients, and found that the risk score model had a correlation with clinical characters using the chi-squared test, including age, isocitrate dehydrogenase (IDH), grade, and methylation of O6-methylguanine-DNA methyltransferase (MGMT) status, which is partly consistent with previous research results [41]. To test the applicability of the risk score model, we used 190 TCGA testing cohort samples to test the model, and the results are consistent with the training set. We then verified the model with the CGGA data validation set (AUC was greater than 0.75), showing that the risk model we constructed could effectively predict the disease prognosis. Although a large number of studies have reported on the prognostic models of glioma, all the studies focused on different points. For example, Du et al. constructed a cancer stem-cell-related model based on CHI3L2, FSTL3, RPA3, RRM2, and YTHDF2 for predicting the prognosis of glioblastoma multiforme [42]. Li et al. suggested that glioma microenvironment-related genes, including LAMB1, FN1, ACTN1, TRIM6, SERPINH1, CYBA, LAIR1, and LILRB2, might serve as potential biomarkers of glioma [43]. Zheng et al. established a pyroptosis-related gene prognostic index for predicting prognosis and for guiding individualized immunotherapy in glioma patients based on CASP3, DPP9, MAPK8, PELP1, and TOMM20 [44]. Compared with the reported glioma prognosis models [45], the AUC value of the ROC curve of our prognosis model is higher. Moreover, our study is the first to construct a prognostic prediction model for glioma patients based on fibroblast-related genes. It has been revealed that cancer-associated fibroblasts can not only promote tumorigenesis and enhance the aggressiveness of cancer cells, but can also introduce chronic inflammation through the production of the pro-inflammatory cytokines responsible for immune tolerance and tumor metastasis [46,47]. Therefore, we believe that identifying fibroblast-related biomarkers and understanding their heterogeneity may provide new directions for the prognosis prediction and treatment of glioma patients. Additionally, to verify whether the risk score can independently predict the survival rate of patients, we conducted an independent prognostic analysis, finding that the risk score was an independent prognostic factor. Furthermore, we developed a nomogram based on age, IDH, grade, MGMT, and risk score to assess the 1-, 3-, and 5-year OS of glioma patients. In current studies, the predictive power of this nomogram outperformed the reported predictive models [48,49]. To explore the mechanisms of the prognostic differences between the high- and low-risk groups, we conducted GSEA. A total of 33 KEGG pathways were clustered into four clusters, including metabolism-related, immune-related, and cancer-related pathways. Therefore, it can be inferred that there were great differences in immunity between the high-risk score and low-risk score groups. At the same time, whether patients can survive longer also depends to some extent on their own immune status.

Immunotherapy refers to the treatment of diseases by artificially enhancing or inhibiting the immune function of the body. There are many immunotherapy methods that are suitable for the treatment of a variety of diseases. Tumor immunotherapy aims to activate the human immune system and kill pathogenic factors (bacteria, fungi, cancer cells, or tumor tissue) by relying on the autoimmune function [50]. Different from surgery, chemotherapy, radiotherapy, and targeted therapy, the target of immunotherapy is not tumor cells and tissues, but the human body’s own immune system. Therefore, reactivating immune cells and reversing the immunosuppressive state of the TME is an important goal of immunotherapy [51]. In this study, we used the microenvironment cell populations-counter (MCP-counter) algorithm to calculate the contents of eight types of immune cells, fibroblasts, and epithelial cells in the sample. After sorting the data and classifying them according to high-risk and low-risk groups, we found that 9 of the 10 types of cells showed significant differences. Immune checkpoint molecules are very important for immune function and have a variety of clinical significance in immunotherapy [52]; therefore, we studied the expression of several key immune checkpoints. It was found that, except for T-cell immunoreceptors with Ig and ITIM domains (TIGIT), immune checkpoint molecules showed significant differences between the high-risk and low-risk groups, and had a higher expression in the high-risk group than in the low-risk group. We used SubMap to predict the response to immunotherapy and found that the high-risk group was more likely to be sensitive to CTLA4 therapy (nominal P and Bonferroni’s correction *p*-value were less than 0.05). As immune checkpoints become clinically targeted, many questions still remain regarding the optimal use of drugs to block the checkpoint pathways [53]. The Genomics of Drug Sensitivity in Cancer (GDSC) project is the largest public resource for information regarding drug sensitivity and the drug response molecular markers of cancer cells. We calculated the IC50 of 37 drugs in the high-risk and low-risk groups, revealing significant differences between the two groups. As previously reported, gefitinib [54], nilotinib [55], lenalidomide [56], axitinib [57], and vorinostat [58] can improve the prognosis of glioma.

DNA methylation is an epigenetic modification in genes that mainly leads to gene transcriptional silencing. It plays an important role in regulating transcription, embryonic development, genomic imprinting, genomic stability, and chromatin structure [59]. To explore the correlation between prognostic gene expression and DNA methylation status, we downloaded the corresponding methylation data from TCGA and proposed the beta value of the CG sites contained in prognostic genes. One gene may correspond to multiple CG sites, and a total of 65 CG sites corresponding to six prognostic genes were found. We analyzed the correlation between the expression of the six prognostic genes and the beta value of the corresponding CG sites of the gene. According to the results, CDK4, NEGR1, MEX3A, and TMEM100 have methylation sites with a significant negative correlation, and the expression of these four genes may be regulated by methylation. Copy number variation (CNV) is caused by genome rearrangement. It generally refers to the increase or decrease in the copy number of large segments of the genome with a length of more than 1 kb, which is mainly manifested in the deletion or duplication at the submicroscopic level. This variation includes both normal polymorphic variation and pathogenic variation. CNV is widely distributed in the human genome, and its mutation rate is much higher than that of single nucleotide polymorphism (SNP). It is one of the most important pathogenic factors of human diseases [60]. We downloaded the copy number variation data of cancer from cBioPortal, including the copy number variation degree of each sample of each gene. To explore whether the prognostic gene is related to the copy number variation, we analyzed the correlation between the copy number variation degree and the prognostic gene expression. The results show that the expression of CDK4 and ANGPTL2 in glioma may be affected by CNV. It is worth noting that genomic CNV may be a novel prognostic biomarker for WHO grade IV glioma patient outcomes [61].

To date, there is still a lack of research on the role that fibroblasts play in the occurrence and development of glioma. In other types of tumors, TAFs can regulate the biological characteristics of tumor cells and stromal cells via cell–cell contact, releasing a variety of regulatory factors, and synthesizing and reshaping ECM, so as to affect the occurrence and development of cancer [33,62]. Through our research and analysis, seven prognostic genes related to TAF in glioma were obtained, and a risk score model was constructed. Based on the model, immunity (immune infiltration, immune checkpoint molecular comparison, and immunotherapy), drug sensitivity, methylation, and copy number analysis were carried out, which linked the prognosis of glioma with tumor biology, providing a theoretical basis and reference value for the research and treatment of glioma. However, there are still several limitations in this study: (a) the results were obtained from the retrospective data of public databases for bioinformatics analysis, and further perspicacity studies are needed to demonstrate their clinical value; (b) the prognostic model was established based on a limited sample size, particularly limited normal samples, and only verified in the CGGA cohort, and thus more datasets and clinical samples are needed to verify the prognostic model; and (c) further experimental research and clinical application research are still needed to further study the role of model genes.

## Figures and Tables

**Table 1:** Clinical characteristics of glioma patients in the training set, testing set, and validation set.

Training Set					Testing Set					Validation Set				
Variable	Total (*n* = 379)	High Risk (*n* = 182)	Low Risk (*n* = 197)	*p*-Value	Variable	Total (*n* = 162)	High Risk (*n* = 77)	Low Risk (*n* = 85)	*p*-Value	Variable	Total (*n* = 463)	High Risk (*n* = 229)	Low Risk (*n* = 234)	*p*-Value
Gender					Gender					Gender				
Female	164 (43.3%)	81 (44.5%)	83 (42.1%)	0.717	Female	67 (41.4%)	31 (40.3%)	36 (42.4%)	0.912	Female	201 (43.4%)	103 (45.0%)	97 (42.4%)	0.638
Male	215 (56.7%)	101 (55.5%)	114 (57.9%)		Male	95 (58.6%)	46 (59.7%)	49 (57.6%)		Male	262 (56.6%)	126 (55.0%)	132 (57.6%)	
Age (years)					Age (years)					Age (years)				
≥60	88 (23.2%)	72 (39.6%)	16 (8.1%)	<0.001	≥60	36 (22.2%)	27 (35.1%)	9 (10.6%)	<0.001	≥60	54 (11.7%)	43 (18.8%)	11 (4.8%)	<0.001
<60	291 (76.8%)	110 (60.4%)	181 (91.9%)		<60	126 (77.8%)	50 (64.9%)	76 (89.4%)		<60	409 (88.3%)	186 (81.2%)	218 (95.2%)	
IDH					IDH					IDH				
IDHmut-codel	103 (27.2%)	16 (8.8%)	87 (44.2%)	<0.001	IDHmut-codel	43 (26.5%)	3 (3.9%)	40 (47.1%)	<0.001	Mutant	250 (54.0%)	51 (22.3%)	196 (85.6%)	<0.001
IDHmut-non-codel	156 (41.2%)	47 (25.8%)	109 (55.3%)		IDHmut-non-codel	57 (35.2%)	13 (16.9%)	44 (51.8%)		Wildtype	213 (46.0%)	178 (77.7%)	33 (14.4%)	
IDHwt	120 (31.7%)	119 (65.4%)	1 (0.5%)		IDHwt	62 (38.3%)	61 (79.2%)	1 (1.2%)		Grade				
Grade					Grade					WHO II	110 (23.8%)	22 (9.6%)	86 (37.6%)	<0.001
G2	149 (39.3%)	38 (20.9%)	111 (56.3%)	<0.001	G2	56 (34.6%)	9 (11.7%)	47 (55.3%)	<0.001	WHO III	176 (38.0%)	60 (26.2%)	114 (49.8%)	
G3	159 (42.0%)	74 (40.7%)	85 (43.1%)		G3	72 (44.4%)	34 (44.2%)	38 (44.7%)		WHO IV	177 (38.2%)	147 (64.2%)	29 (12.7%)	
G4	71 (18.7%)	70 (38.5%)	1 (0.5%)		G4	34 (21.0%)	34 (44.2%)	0 (0%)		MGMT				
MGMT					MGMT					Methylated	273 (59.0%)	134 (58.5%)	137 (59.8%)	0.849
Methylated	285 (75.2%)	101 (55.5%)	184 (93.4%)	<0.001	Methylated	118 (72.8%)	36 (46.8%)	82 (96.5%)	<0.001	Unmethylated	190 (41.0%)	95 (41.5%)	92 (40.2%)	
Unmethylated	94 (24.8%)	81 (44.5%)	13 (6.6%)		Unmethylated	44 (27.2%)	41 (53.2%)	3 (3.5%)		Histology				
										A	53 (11.4%)	13 (5.7%)	39 (17.0%)	<0.001
										AA	63 (13.6%)	23 (10.0%)	40 (17.5%)	
										AO	31 (6.7%)	4 (1.7%)	27 (11.8%)	
										AOA	4 (0.9%)	3 (1.3%)	1 (0.4%)	
										GBM	108 (23.3%)	87 (38.0%)	21 (9.2%)	
										O	29 (6.3%)	0 (0%)	29 (12.7%)	
										OA	2 (0.4%)	0 (0%)	2 (0.9%)	
										rA	16 (3.5%)	5 (2.2%)	10 (4.4%)	
										rAA	55 (11.9%)	26 (11.4%)	28 (12.2%)	
										rAO	23 (5.0%)	4 (1.7%)	18 (7.9%)	
										rGBM	69 (14.9%)	60 (26.2%)	8 (3.5%)	
										rO	10 (2.2%)	4 (1.7%)	6 (2.6%)	
										Radio_status				
										Radio_treated	366 (79.0%)	188 (82.1%)	174 (76.0%)	0.136
										Radio_untreated	97 (21.0%)	41 (17.9%)	55 (24.0%)	
										Chemo_status				
										Chemo_treated	358 (77.3%)	187 (81.7%)	169 (73.8%)	0.0562
										Chemo_untreated	105 (22.7%)	42 (18.3%)	60 (26.2%)	

## Data Availability

Publicly available datasets were analyzed in this study. These data can be found here: https://portal.gdc.cancer.gov (accessed on 7 February 2022), http://www.cgga.org.cn (accessed on 31 May 2019), and http://www.gsea-msigdb.org/gsea/msigdb (accessed on 6 February 2022).

## Supplementary Materials

The following supporting information can be downloaded at: https://www.mdpi.com/article/10.3390/biom12111598/s1. Figure S1. Data processing pipeline. Figure S2. Kaplan–Meier survival curve of 57 DEFRGs associated with glioma survival. Figure S3. (A) Correlation between risk score and clinical features in the training cohort. (B) Correlation between risk score and clinical features in the testing cohort. (C) Correlation between risk score and clinical features in the validation cohort. Figure S4. Clustering of common GO items based on the scores calculated using the ssGSEA method. Figure S5. Correlation scatter plot of the correlations between the expression of prognostic genes and different cg sites (without significantly negatively correlations): (A) CDK4; (B) NEGR1; (C) MEX3A; (D) TMEM100; (E) PBK; and (F) SLC2A3. Table S1. Clinical data and survival information of 635 glioma samples from the TCGA database. Table S2. Clinical information of 657 glioma samples from the CGGA database. Table S3. Detailed DEG information between the glioma and normal samples in the TCGA cohort. Table S4. Gene list of FRGs and DEFRGs. Table S5. Functional enrichment result of DEFRGs. Table S6. The *p*-value of the K–M curve of DEFRGs. Table S7. The 47 DEFRGs related to the prognosis of glioma patients. Table S8. The model genes obtained by LASSO regression algorithm. Table S9. The GSEA result of high- and low-risk groups. Table S10. The GSEA result of patients with survival times longer and shorter than 5 years. Table S11. Common GO items and KEGG pathways enriched by high- and low-risk groups, and patients with survival times longer and shorter than 5 years. Table S12. Correlation between the expression of prognostic genes and the corresponding methylation sites.
