# Supplementary figures and images for: Identification of a Fibroblast-Related Prognostic Model in Glioma Based on Bioinformatics Methods
Source: Biomolecules. 2022 Oct 30;12(11):1598. doi: 10.3390/biom12111598 (PMC9687522; doi:10.3390/biom12111598)

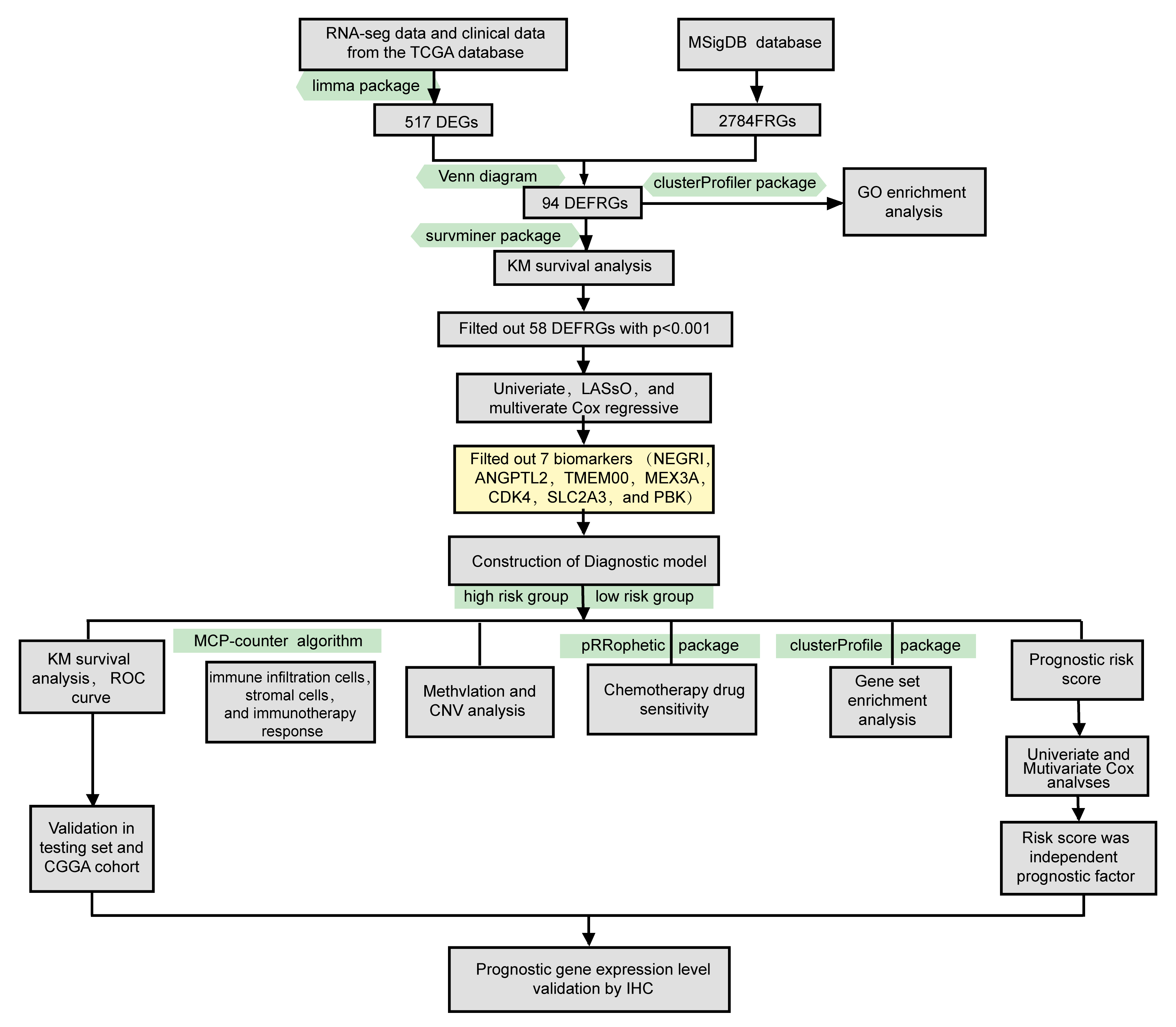

Supplement: Supplementary file 1 [file biomolecules-12-01598-s001.zip › Supplementary Figure S1.TIF]

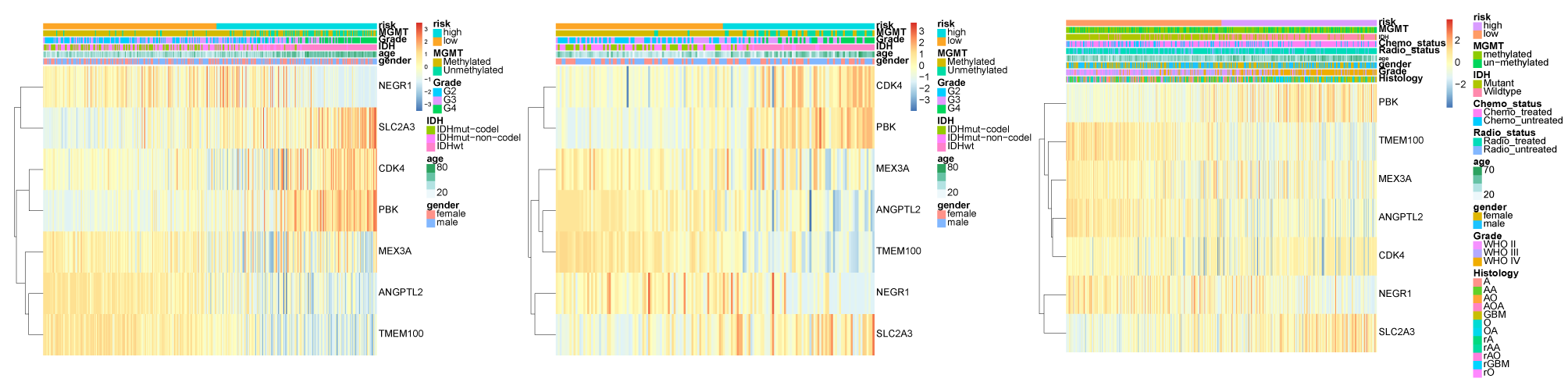

Supplement: Supplementary file 1 [file biomolecules-12-01598-s001.zip › Supplementary Figure S3.TIF]

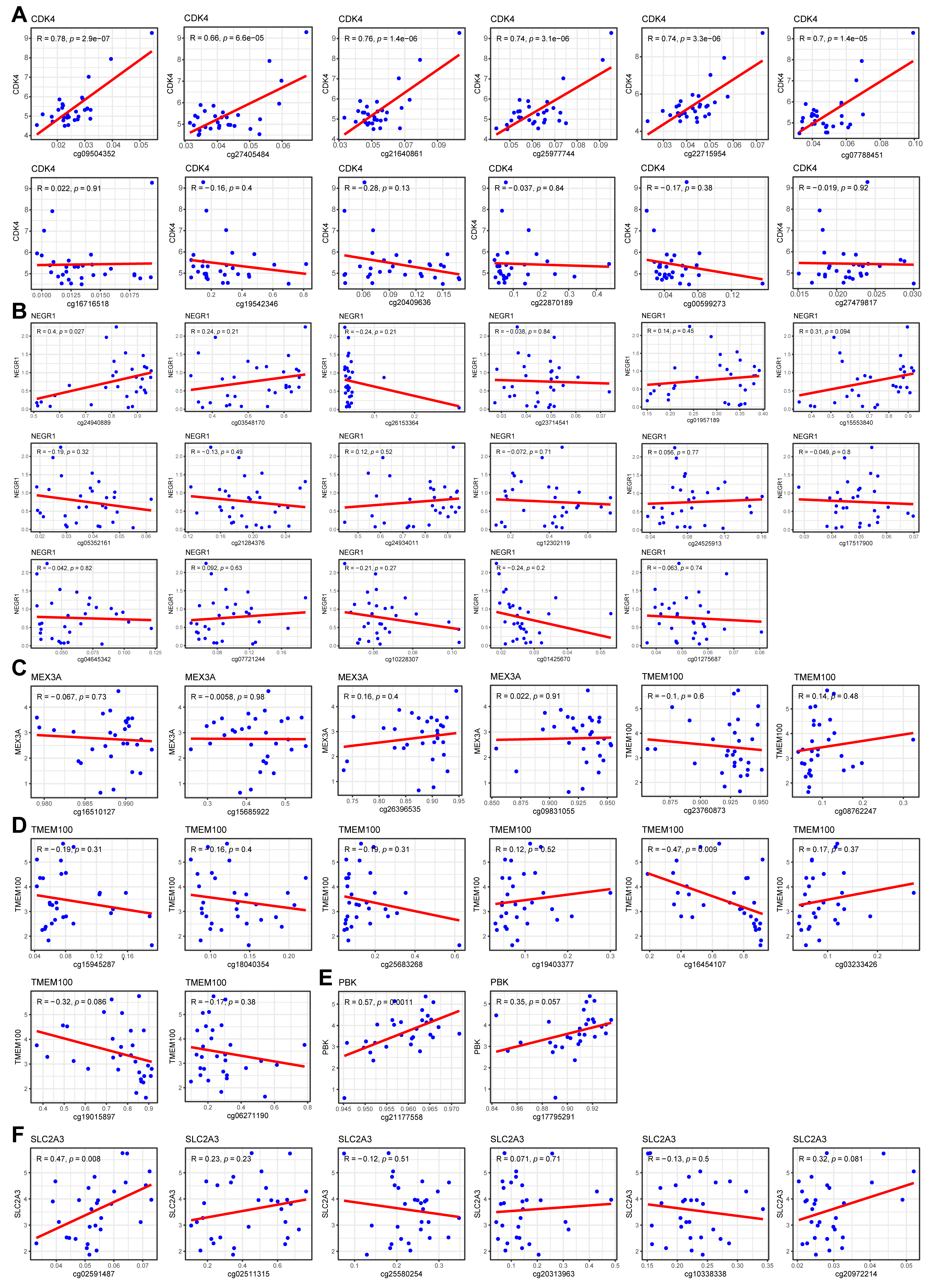

Supplement: Supplementary file 1 [file biomolecules-12-01598-s001.zip › Supplementary Figure S5.TIF]
